# Enhanced efficacy of *Helicoverpa armigera* nucleopolyhedrovirus against *Spodoptera litura* larvae using zeolite and chitosan nanoparticle formulations

**DOI:** 10.1016/j.virusres.2025.199614

**Published:** 2025-08-05

**Authors:** Mia Miranti, Iqbal Nur Iskandar, Melanie Melanie, Desak Made Malini, Camelia Panatarani, I Made Joni, Dedat Prismantoro, Febri Doni, Ravindra Chandra Joshi, Wawan Hermawan

**Affiliations:** a Department of Biology, Faculty of Mathematics and Natural Sciences, Universitas Padjadjaran, Jatinangor 45363, West Java, Indonesia; b Functional Nano Powder University Centre of Excellence, Faculty of Mathematics and Natural Sciences, Universitas Padjadjaran, Jatinangor 45363, West Java, Indonesia; c Department of Physics, Faculty of Mathematics and Natural Sciences, Universitas Padjadjaran, Jatinangor 45363, West Java, Indonesia; d Department of Global Development, Cornell University, Ithaca, NY 14853, USA; e Philippine Rice Research Institute, Maligaya, Science City of Muñoz, Nueva Ecija 3119, Philippines

**Keywords:** Formulation, *Ha*NPV_1_, Chitosan nanoparticle, Mortality, *S. litura*, Lethal time, Zeolite nanoparticle

## Abstract

*Helicoverpa armigera* nucleopolyhedrovirus (HearNPV1) shows considerable potential as a sustainable biological control agent against insect pests. Enhancing its efficacy through formulation with appropriate carrier materials is critical for improving pest management strategies. This study evaluated the potential of chitosan and zeolite nanoparticles as delivery vehicles for HearNPV1, assessing their effects on mortality and lethal time in second-instar *Spodoptera litura* larvae. A single-factor randomized block design with three replications was used. The treatments consisted of HearNPV1 formulated with various concentrations (0.125 % to 0.5 %) of either chitosan or zeolite nanoparticles. Larval mortality and lethal time (LT) were recorded and analyzed using one-way ANOVA, with significant differences further assessed by Duncan's multiple range test (α=0.05). Results showed that formulating HearNPV1 with either chitosan or zeolite nanoparticles significantly increased larval mortality. Notably, even the lowest concentration tested (0.125 %) of both chitosan and zeolite nanoparticles achieved 66.67 % larval mortality within seven days post-infection. However, no significant differences in lethal time were observed among treatments, regardless of nanoparticle type or concentration. Larvae exposed to virus–nanoparticle formulations exhibited lethal times ranging from 3.0 to 4.67 days on average—similar to the duration observed in larvae infected with HearNPV1 alone. In conclusion, both chitosan and zeolite nanoparticles at 0.125 % concentrations effectively enhanced HearNPV1-induced mortality in *S. litura* larvae, although they did not shorten the time to death. These findings underscore the potential for nanoparticle-based HearNPV1 formulations to improve biological pest control, warranting further investigation into their practical application and mechanisms of action.

## Highlights

• HearNPV1 was formulated with chitosan and zeolite nanoparticles for pest control.
• Nanoparticle formulations significantly increased *Spodoptera litura* larval mortality.
• The lowest nanoparticle concentration (0.125 %) caused 66.67 % mortality in 7 days.
• Lethal time (LT) remained unchanged across nanoparticle types and concentrations.
• Results support nanoparticle-based viral biopesticides for enhanced field efficacy.

## 1 Introduction

Entomopathogenic viruses are increasingly recognized for their high specificity toward target insect species (Kalha et al., 2014) and their natural capacity to trigger epizootics in insect populations (Anggraini et al., 2022). Among these, baculoviruses—which include occlusion body-forming nucleopolyhedroviruses (NPVs) and highly host-specific granuloviruses (GVs) —have been extensively studied and established as effective biological control agents against a wide range of economically important insect pests. Importantly, they are non-pathogenic to humans (Afolami and Oladunmoye, 2017), making them environmentally safe alternatives to chemical pesticides. Their potential has been validated through numerous field trials. Baculoviruses produce polyhedral occlusion bodies (OBs) that confer protection against adverse environmental conditions, thereby enhancing their persistence in the field (Williams et al., 2017; Jia et al., 2023). However, despite these advantages, their field efficacy is often limited by factors such as a relatively narrow host range in some isolates and a slow speed of kill (Anggraini et al., 2022).

The *Helicoverpa armigera* nucleopolyhedrovirus (HearNPV) was originally isolated from *H. armigera* (Hübner) larval cadavers (Arrizubieta et al., 2022; Miranti et al., 2023). Interestingly, this virus can also be propagated in *Spodoptera litura* (Fabricius) larvae, which serve as a viable alternative host (Miranti et al., 2023). The subcultured HearNPV has demonstrated infectivity not only in *S. litura* but also in *Crocidolomia pavonana* (Fabricius) (Miranti et al., 2023; Prasetio et al., 2020). Moreover, HearNPV has been shown to infect other heterologous hosts, such as *Spodoptera frugiperda* (J.E. Smith) and *Mamestra brassicae* (Linnaeus) (Arrizubieta et al., 2022), suggesting that its host range may be broader than previously assumed.

The development of advanced formulations using suitable carrier materials can significantly enhance the field performance and persistence of viral biopesticides, thereby increasing their effectiveness in inducing mortality in target insect pests (Abd-Alla et al., 2020). Considerable research has been conducted to identify effective additives, including synthetic agents such as spreaders, surfactants, dyes, optical brighteners, and lignin derivatives, as well as various natural substances (Anggraini et al., 2022; Bandi et al., 2019; Wilson et al., 2020). Natural materials explored for their carrier potential include moringa, cacao, green tea, lantana, benzopurpurine, charcoal, iron dioxide, benzimidazole, kaolinite, and bentonite (Baranwal et al., 2022; Çakmak et al., 2021; Melanie et al., 2022).

Transforming these natural materials into nanoparticle form has been shown to enhance their efficacy as carriers. For example, zeolite nanoparticles have been reported to reduce the lethal time by 60.4 % compared to traditional zeolite microparticles when used in virus formulations (Miranti et al., 2023). Nanoparticles are generally defined as materials with dimensions between 1 and 100 nm ( Ajdary et al., 2018; Sharma et al., 2015), although some definitions broaden this range to include particles smaller than 1000 nm (Perlatti et al., 2013). They can be classified as either naturally occurring or synthetic, with the former often being less toxic due to their biogenic origins (Sharma et al., 2015). Additional classification schemes consider factors such as particle shape (e.g., tubes, spheres), electrical and chemical properties, and origin (e.g., natural derived, like viruses, or synthetic) (Ajdary et al., 2018).

Carrier materials are essential in viral formulations, primarily because they protect viral particles from degradation, especially by UV radiation. The physical form of the formulation also affects its stability. For instance, dry formulations of HearNPV tend to exhibit greater stability than aqueous ones, likely due to lower microbial contamination and reduced degradation (Mane et al., 2016; Quiroga-Cubides et al., 2022). Ideally, carrier materials should not only protect the virus but also enhance its virulence, including reducing the time required to kill the host insect. Among potential carriers, zeolite and chitosan nanoparticles are particularly promising.

Chitosan, a natural polymer derived from chitin (commonly sourced from crustacean shell waste), is biodegradable, renewable, and non-toxic (Kou et al., 2021; Ureña-Saborío et al., 2017). Its nanoparticle form offers improved performance, including enhanced efficiency, bioavailability, and specificity (El-Morsy et al., 2023; Ureña-Saborío et al., 2017). Zeolites, on the other hand, are microporous crystalline materials with high surface areas and well-defined structures (Jena et al., 2023; Kadja et al., 2022; Mohsin et al., 2023). Their functional properties can be significantly enhanced by nanoscale modification through techniques such as nanoparticle doping, which incorporates nanosized particles into the zeolite matrix (Ruíz-Baltazar, 2024). This process increases the number of active sites for molecular interaction and improves adsorption capacity, thus boosting overall functionality (Ravindran et al., 2024; Wang et al., 2023).

Chitosan nanoparticles have been successfully employed in various applications, including the delivery of *Fusarium* species (El-Morsy et al., 2023), probiotic encapsulation (Riseh et al., 2022), and the formulation of *Bacillus thuringiensis* (Berliner) for insect control (Duraimurugan et al., 2024). In addition to their role as carriers, chitosan nanoparticles also exhibit antimicrobial activity (Divya and Jisha, 2018). Similarly, zeolite nanoparticles have been used to encapsulate entomopathogenic fungi, such as *Metarhizium anisopliae* (Mechnikov) Sorokin (Ilhami et al., 2020) and as virus carriers (Prasetyo et al., 2020). They are also being explored for antimicrobial applications (Qi et al., 2021; Wang et al., 2017) and drug delivery (Pandya et al., 2024).

Given these promising characteristics, chitosan and zeolite nanoparticles represent strong candidates for use as virus carrier materials in biopesticide formulations. This study hypothesizes that incorporating either chitosan or zeolite nanoparticles into viral formulations can enhance virus efficacy, potentially producing a synergistic bioinsecticidal effect against target pests. Therefore, *S. litura* larvae will be used as model to evaluate the effects of these nanoparticle-based carriers on larval mortality and lethal time.

## 2 Materials and methods

### 2.1 Experimental design

This study employed a single-factor randomized block design with three replications. Treatments consisted of a control (HearNPV1 suspension only) and virus formulations incorporating either chitosan nanoparticles or zeolite nanoparticles as carrier materials. Each nanoparticle type was tested at three concentrations: 0.5 %, 0.25 %, and 0.125 % The primary response variables measured were larval mortality ( %) and lethal time (days).

### 2.2 Larval preparation

A starter colony of *S. litura* larvae was obtained from the Indonesian Vegetables Research Institute, located at 517 Tangkuban Parahu, Lembang, West Java, Indonesia (latitude: −6.801655, longitude: 107.649076). The larvae were reared at the Laboratory of Biomolecular and Biosystem, Universitas Padjadjaran, Indonesia, under controlled conditions using a rearing cabinet (Lutron HT-3006A) set at 28 ± 1 °C, 80 ± 5 % relative humidity, and a 12:12 h light:dark photoperiod. Larvae were fed insecticide-free cabbage leaves (Miranti et al., 2023).

Rearing continued through the complete life cycle. Larvae were reared to pupation, and pupae were transferred to containers with soil as a pupation substrate. After adult emergence, moths were placed in oviposition cages equipped with ovitraps for egg collection and supplied with a 10 % honey solution as a food source. Eggs were transferred to clean, sterile containers and maintained until hatching into first-instar larvae (Sharma et al., 2022).

Developmental stages were monitored by observing molting events and measuring head capsule width to confirm instar transitions. Each instar was housed separately to prevent cannibalism and ensure developmental consistency. Third-instar larvae were selected for use in all bioassays.

### 2.3 Viral preparation

The *Helicoverpa armigera* nucleopolyhedrovirus (HearNPV1) used in this study was originally isolated from infected *H. armigera* larvae collected at the Laboratory of Applied Microbiology, Universitas Padjadjaran, Indonesia. The virus was subsequently propagated in *S. litura* larvae, used as an alternative host.

For viral amplification, fourth-instar *S. litura* larvae were fed cabbage leaves inoculated with viral occlusion bodies (OBs). OBs were extracted and purified muslin cloth filtration, followed by centrifugation and washing, as described by Miranti et al. (2023).

Reagents used for virus purification included sodium dodecyl sulphate (Merck KGaA, Darmstadt, Germany), Tris buffer (Merck KGaA, Darmstadt, Germany), sodium chloride (Merck KGaA, Darmstadt, Germany), Aquadest (Bratachem, Jakarta, Indonesia), and sodium azide (Merck KGaA, Darmstadt, Germany). For virus purification, infected larval cadavers were collected and homogenized in a 1:1 solution of 0.1 % SDS and 1 mM Tris buffer (pH 7.6), following the method of O'Reilly et al. (1994). The resulting suspension from the ground larvae was then filtered through cotton cloth The ground tissues containing the virus occlusion bodies (OBs) were centrifuged at 3500 rpm for 10 min at room temperature. After discarding the supernatant, the pellet was resuspended in 1 mL of 0.1 % SDS and 1 mL of 1 mM Tris buffer, vortexed, and centrifuged again under the same conditions.

The final OB pellet was suspended in 0.5 mL of 0.1 % SDS and 0.1 mM Tris buffer per larva using pipetting or gentle vortexing. OB concentration was determined by mixing 0.1 mL of the virus suspension with 0.9 mL of a 1:1 mixture of 1 mM Tris buffer (pH 7.6) and 0.1 % SDS, followed by counting with a Neubauer hemocytometer under a light microscope at 400× magnification. OBs were identified by their characteristic cuboidal shape and green color. Their morphology was further confirmed by scanning electron microscopy (SEM; JEOL JSM-6360LA) at 10,000× magnification. The final virus stock contained 2.6 × 10⁹ OBs/mL. A working concentration of 4 × 10⁷ OBs/mL was used in the bioassays.

### 2.4 Nanoparticles preparation

Natural chitosan and zeolite powders were used as carrier materials and converted into nanoparticles using a beads-milling method—a top-down mechanical process that reduces particle size (Joni et al., 2012; Rochima et al., 2018). To begin, a slurry of each material was prepared by mixing the powder with distilled water, followed by magnetic stirring for 30 min. Polyethylene glycol (PEG 400) was added as a dispersing agent after 15 min of stirring, at a ratio of 150 wt fractions relative to the chitosan or zeolite powder. The milling system consisted of a 250 mL vessel, a pump, and a mixing tank, with the vessel filled to 70 % of its capacity using zirconia beads. The prepared slurry was recirculated through the vessel at a flow rate of 8 L/min, where an impeller rotating at 4070 rpm agitated the beads to disrupt particle aggregates and prevent agglomeration. The mixture then passed into a separation chamber where centrifugal force removed the zirconia beads before the suspension was returned to the dispersing zone. A water jacket system was used to maintain a constant temperature, and the vessel was sealed throughout the process to ensure stability.

The optimal conditions for nanoparticle synthesis included a starting material concentration of 0.1 wt% in the slurry and a milling duration of 120 min. For chitosan nanoparticles, 1 vol% acetic acid was added after milling to induce a positive surface charge. The particle size distribution of the resulting chitosan and zeolite nanoparticles was measured using dynamic light scattering (DLS) with a Delsa™ Nano particle size analyzer (Beckman Coulter, USA). Final stock suspensions of both nanoparticle types were prepared in deionized water at a concentration of 1 % (w/v) for use in subsequent experiments.

### 2.5 Characterizations of chitosan and zeolite nanoparticles

The particle size distribution and polydispersity index (PI) of chitosan and zeolite nanoparticles were measured using particle size analysis (PSA) based on dynamic light scattering (DLS). Samples were dispersed in deionized water and sonicated for 10 min to ensure uniform suspension. Measurements were conducted at room temperature using a standard PSA instrument, and average particle size and PI values were recorded to evaluate the effects of the beads-milling process.

### 2.6 Virus formulation in nanoparticles

Virus formulations were prepared by mixing 15 mL of stock virus suspension with 1000 mL of either chitosan or zeolite nanoparticle suspension. The pH of each formulation was adjusted to approximately 6.9–7.1 to ensure stability and compatibility with the virus. These nanoparticle-virus formulations, intended for larval infection assays, had a final virus concentration of 4 × 10⁷ OBs/mL. A control treatment consisted of the virus suspension without any nanoparticles, also maintained at the same concentration.

### 2.7 Bioassay tests

Third-instar larvae of *S. litura* were used for the bioassay experiments, with each treatment replicated three times. For each treatment, 0.30 mL of the prepared virus formulation (either nanoparticle-based or control) was evenly applied to a 10 cm diameter cabbage leaf free of synthetic insecticides. Prior to exposure, larvae were starved for 3 h to encourage feeding. Five larvae were then placed in each container with the treated leaf. After a 24-hour exposure period, the treated leaves were removed and replaced daily with fresh, untreated cabbage leaves for the remainder of the 7-day trial.

Larval mortality was monitored and recorded daily. Mortality ( %) was calculated as the proportion of dead larvae relative to the total number exposed per treatment. Lethal time (LT) was defined as the number of days from initial exposure to the virus until the death of each larva.

### 2.8 Data analysis

The total number of polyhedral (OBs) was calculated using the formula as follows:

(1) $$\frac{N}{V}\times P\times 10^{3}$$

Where,

*N* = number of polyhedral counted;

*V* = area counted (Neubauer counting chamber);

*P* = dilution factor.

The average lethal time (W) was calculated using the formula in Eq. (1) for 7 days of observation, according to Prasetio et al. (2020) as follows:

(2) $$W=\frac{\sum W_{i}\times Z_{i}\;}{Y}$$

Where,

*W* = average lethal time (days);

W_i_ = lethal time on day i (and not day 1);

Z_i_ = number of insects that died on day i (and not day 1);

*Y* = total number of insects that died during the observation period.

The data were collected and organized using Microsoft Excel (Version 16, Microsoft Corporation, Redmond, WA, USA). Data were analyzed using one-way analysis of variance (ANOVA) in SPSS software version 26 (IBM Corp., Armonk, NY, USA). When significant differences were detected (*p* ≤ 0.05), Duncan’s Multiple Range Test (DMRT) was applied for post hoc comparisons. Results are presented as mean ± standard error (SE).

## 3 Results

### 3.1 The structure of *hear*npv subculture

**Fig. 1:**
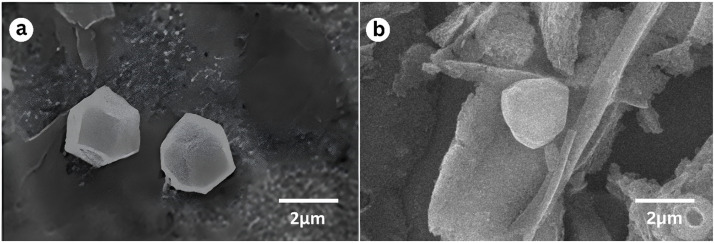
Morphological structure of HearNPV occlusion bodies: (a) original HearNPV and (b) subcultured HearNPV in *S. litura*, observed at 10,000× magnification.

The structure of occlusion bodies (OBs) from the *Helicoverpa armigera* nucleopolyhedrovirus (HearNPV) subcultured in the alternate host, *S. litura*, was examined using scanning electron microscopy (SEM) at 10,000× magnification. The resulting morphology was compared to that of OBs propagated in its primary host, *H. armigera*, as presented in Fig. 1. Observation revealed that OBs from the subcultured virus in *S. litura* exhibited a more rounded shape (Fig. 1b), whereas OBs from the original host (*H. armigera*) displayed a distinct hexagonal morphology (Fig. 1a).

### 3.2 Particle size distribution before and after beads-milling

**Fig. 2:**
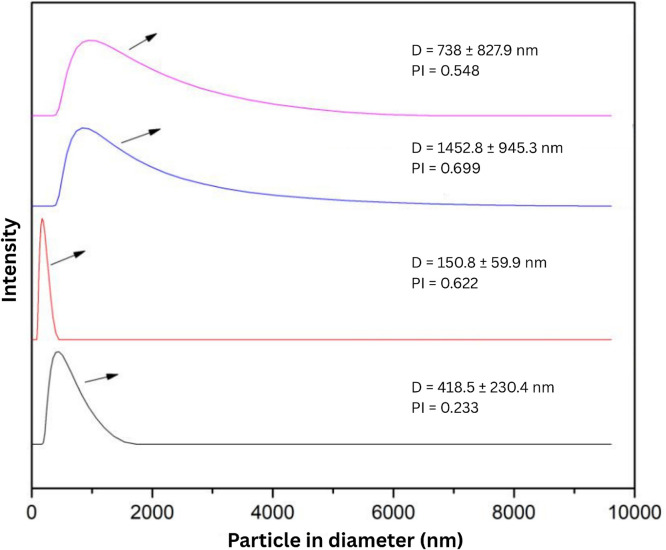
Particle size analysis of nanoparticles: (a) chitosan after bead-milling, (b) chitosan before bead-milling, (c) zeolite after bead-milling, and (d) zeolite before bead-milling.

The particle size distribution of zeolite and chitosan was significantly affected by the beads-milling process. For zeolite, the average particle diameter was reduced from 418.5 ± 230.4 nm (Polydispersity Index [PI] = 0.233) before milling to 150 ± 59.9 nm after milling. Post-milling, the PI increased to 0.622, indicating a broader size distribution. Chitosan particles also exhibited a size reduction from 1452.8 ± 945.3 nm (PI = 0.699) to 738 ± 827.9 nm after milling. In contrast to zeolite, the PI for chitosan decreased to 0.548 after milling, indicating improved particle size uniformity. Despite the reduction, the average particle size of 738 nm remains within the nanoparticle classification (<1000 nm). These changes in size and distribution are visually represented in Fig. 2.

### 3.3 *Hear*NPV subculture formulation test in zeolite and chitosan nanoparticle carrier materials of *S. litura* larva mortality

The analysis of variance (ANOVA) on larval mortality data showed significant differences among treatments. Therefore, Duncan’s Multiple Range Test (DMRT) was applied to identify pairwise differences at *p* < 0.05. Additionally, 95 % confidence intervals were calculated for each treatment to provide a clearer estimate of variability and support interpretation of the observed differences. The results are summarized in Table 1.

**Table 1:** Mortality of larvae *S. litura* infected with the HearNPV formulated with chitosan and zeolite nanoparticles.

No.	Treatments	Average mortality ( %)
1.	Control (*Hear*NPV_1_)	20 ± 0.00^b^
2.	Chitosan Nanoparticles 0.5 % + *Hear*NPV_1_	26.33 ± 11.55^b^
3.	Chitosan Nanoparticles 0.25 % + *Hear*NPV_1_	46.67 ± 11.55^ab^
4.	Chitosan Nanoparticles 0.125 % + *Hear*NPV_1_	66.67 ± 30.55^a^
5.	Zeolite Nanoparticles 0.5 % + *Hear*NPV_1_	33.33 ± 11.55^ab^
6.	Zeolite Nanoparticles 0.25 % + *Hear*NPV_1_	60 ± 34.64^a^
7.	Zeolite Nanoparticles 0.125 % + *Hear*NPV_1_	66.67 ± 41.63^a^

Note: Mean numbers followed by the same lowercase letter in the same column are not significantly different according to DMRT test (*p* < 0.05).

The formulations containing either chitosan or zeolite nanoparticles showed significantly higher larval mortality (ranging from 26.33 % to 66.67 %) compared to the control group, which had the lowest mortality rate (20 %). No significant differences were found between the concentrations tested (0.125 % to 0.5 %) for either carrier material, indicating that even the lowest concentration (0.125 %) was sufficient to enhance mortality effectively.

**Fig. 3:**
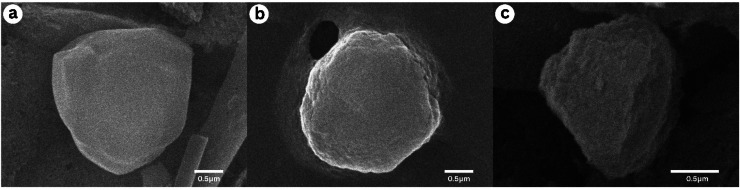
(a) Structure of the native viral occlusion body; (b) viral occlusion body encapsulated in a 0.125 % chitosan nanoparticle formulation; and (c) viral occlusion body encapsulated in a 0.125 % zeolite nanoparticle formulation.

The structural integrity of viral occlusion bodies (OBs) under nanoparticle formulations was examined via SEM. As shown in Fig. 3, the OBs from the virus-only control (Fig. 3a) exhibited smooth surfaces, whereas OBs formulated with 0.125 % chitosan (Fig. 3b) and zeolite (Fig. 3c) nanoparticles showed visibly rougher textures. This suggests that nanoparticle interaction may physically alter OB surfaces during encapsulation, potentially influencing viral bioactivity.

### 3.4 *Hear*NPV subculture formulation test in zeolite and chitosan nanoparticle carrier materials of *S. litura* larva lethal time

The analysis of variance (ANOVA) for larval lethal time did not reveal significant differences among the treatments; thus, Duncan’s multiple range test was not conducted. The results are summarized in Table 2. The average lethal time across treatments ranged from 3.0 to 4.67 days. Although no statistical significance was observed, higher concentrations of nanoparticles appeared to correlate with a slightly faster time to larval death.

**Tabel 2:** The lethal time of *S. litura* larvae after being infected with the virus formula.

No.	Treatments	Average lethal time (days)
1.	Control (*Hear*NPV_1_)	4.33 ± 1.53
2.	Chitosan Nanoparticles 0.5 % + *Hear*NPV_1_	3.5 ± 1.80
3.	Chitosan Nanoparticles 0.25 % + *Hear*NPV_1_	4.16 ± 1.26
4.	Chitosan Nanoparticles 0.125 % + *Hear*NPV_1_	4.67 ± 1.15
5.	Zeolite Nanoparticles 0.5 % + *Hear*NPV_1_	3.0 ± 2.00
6.	Zeolite Nanoparticles 0.25 % + *Hear*NPV_1_	4.56 ± 1.83

## 4 Discussion

The structural differences observed between HearNPV occlusion bodies (OBs) derived from *H. armigera* and those subcultured in *S. litura* suggest morphological adaptation depending on the propagation host. Despite the more rounded appearance of the OBs in the subcultured virus, infectivity remained high. In fact, *S. litura* larvae infected with the subcultured HearNPV exhibited a mortality rate of 86.4 % (Miranti et al., 2023), indicating that the altered OB structure did not diminish the virus's bioefficacy. This result underscores the robustness of HearNPV's pathogenicity, even when propagated in an alternate host species.

Beads-milling effectively reduced the particle sizes of both zeolite and chitosan, transforming them into the nanoparticle range. The decreased particle diameter of zeolite accompanied by a higher PI value suggests that milling increased size variability, making the formulation more polydisperse. Conversely, the reduction in both particle size and PI for chitosan nanoparticles indicates a more uniform and stable formulation post-milling. The classification of these materials as nanoparticles is supported by size thresholds described in literature, where particles below 1000 nm qualify as nanoparticles (Peratti, 2013; Shamzhy et al., 2019). The contrasting PI trends between zeolite and chitosan highlight the importance of tailoring the milling process to each material’s properties to achieve optimal formulation characteristics for delivery applications.

The data indicate that nanoparticle-assisted formulations of HearNPV1 significantly improve mortality in *S. litura* larvae compared to the virus-only treatment. The 0.125 % formulations of both chitosan and zeolite nanoparticles achieved mortality rates as high as 66.67 %, a notable enhancement over the 20 % mortality observed in the control. These findings suggest that even low concentrations of nanoparticle carriers may contribute to enhanced larval mortality when used in combination with HearNPV. However, no significant increase in mortality was observed with higher nanoparticle concentrations, which is consistent with previous studies. For instance, Duraimurugan et al. (2024) also observed that lower concentrations of chitosan nanoparticles encapsulating *B. thuringiensis* resulted in higher larval mortality (up to 70 %) in *S. litura*. A potential explanation for this phenomenon lies in nanoparticle aggregation and precipitation at higher concentrations. Increased polymer linkages and hydrophobic interactions may reduce nanoparticle dispersion and bioavailability, ultimately diminishing efficacy (Wang and Xiong, 2019).

Fig. 3 provides further insight by illustrating the OBs’ surface morphology. Encapsulation with nanoparticles resulted in rougher OB surfaces (Figs. 3b and 3c) compared to the virus-only OBs (Fig. 3a). This altered surface structure could influence virus-host interactions. Although some studies report that damage to OBs may reduce viral infectivity (Abdelkhalek et al., 2021), others highlight the intrinsic antiviral properties of nanoparticles due to their unique physicochemical characteristics (Hussain et al., 2022).

A primary mechanism proposed to explain how nanoparticles enhance antiviral effects is their ability to bind to the surface of OBs, potentially altering the virus–host interaction. This binding may interfere with the virus's ability to attach to and infect host cells by modifying surface charge, blocking receptor access, or changing the physical structure of the OBs (Zhou et al., 2021). Such interactions could enhance viral stability, prolong persistence on plant surfaces, or improve adherence to the insect midgut, thereby increasing the chances of successful infection. These effects may collectively contribute to the observed enhancement in larval mortality following exposure to virus–nanoparticle formulations.

The observed trend of reduced lethal time at higher nanoparticle concentrations, despite the lack of statistical significance, suggest a possible synergistic interaction between the nanoparticle carriers and viral infectivity. One plausible explanation is that elevated nanoparticle levels may facilitate more efficient uptake by insect cells, potentially accelerating physiological stress response. This hypothesis is supported by previous findings, such as those of Mir et al. (2020), who demonstrated that zinc oxide nanoparticles (ZnO NPs) can penetrate the insect gut barrier and exert cytotoxic effects. If chitosan and zeolite nanoparticles in the present study behave similarly, the combined effects of viral infection and nanoparticle-induced stress could contribute to earlier larval mortality. This implies that the observed enhancement is not necessarily due to increased mortality percentages, but rather to a reduction in the time required to reach lethal outcomes. These findings contribute to current research by evaluating two types of nanoparticle carriers across different concentrations to determine their role in enhancing viral effectiveness without compromising occlusion body integrity.

Although further studies are needed to validate field applicability, the observed reduction in time to larval mortality suggests a possible synergistic interaction between the nanoparticle carriers and viral infectivity. Such interactions could have practical implications in pest management by potentially reducing exposure time and minimizing crop damage. A summary of previously reported nanoparticle-assisted biopesticide formulations is provided in Table 3 to contextualize the performance of the present study within the broader research landscape. Given the increasing global scrutiny of nanomaterials in agroecosystems, it is important to emphasize that the present study employed chitosan and zeolite nanoparticles solely as delivery agents under laboratory conditions (Tripathi et al., 2023; Zhang et al., 2025). Future investigations should include mechanistic, histopathological, and environmental assessments to better understand nanoparticle-virus interactions and ensure their safe and responsible use in field applications.

**Table 3:** Comparative studies on nanoparticle-assisted formulations of microbial biopesticides relevant to the present work.

Species names	Nanoparticles	Mechanisms	Target organisms	Mortality ( %)	Lethal time (days)	References
HearNPV	Nanozeolite and nanochitosan	Delivery carrier	*S. litura*	66.67	3	This study
*M. anisopliae*	Nanozeolite	Encapsulation	*C. pavonana*	92.5	1.08	Ilhami et al., 2020
*B. thuringiensis*	Nanochitosan	Microencapsulation	*S. litura*	70	> 7	Duraimurugan et al., 2024
*Bacillus* BMI strain B-458	Chitosan nanoparticles	Delivery carrier	*Radopholus similis*	100	3	Ureña-Saborío et al., 2017

## 5 Conclusions

This study revealed a clear pattern in *S. litura* larval mortality and lethal time in response to different concentrations of chitosan and zeolite nanoparticles. The highest mortality was observed at lower concentrations (e.g., 0.125 %), suggesting a synergistic interaction between the nanoparticles and the virus. At these levels, the nanoparticles likely do not compromise viral integrity but instead enhance viral efficacy through mechanisms such as improved stability, increased adhesion to host tissues, or facilitated cellular uptake.

In contrast, higher nanoparticle concentrations were associated with a more rapid lethal time, despite the possibility of some adverse effects on viral particles. This accelerated response may result from an increased nanoparticle load promoting faster cellular entry and earlier onset of pathogenic effects—either by supporting the activity of remaining viable virions or through additive toxic effects. Based on these findings, using low concentrations of chitosan or zeolite nanoparticles appears to be an optimal, cost-effective strategy for biopesticide delivery. Future studies should investigate their environmental persistence and non-target impacts to ensure the safe and sustainable use of nanoparticle-based biopesticides in agroecosystems.

## CRediT authorship contribution statement

**Mia Miranti:** Writing – review & editing, Writing – original draft, Supervision, Project administration, Methodology, Investigation, Funding acquisition, Formal analysis, Data curation. **Iqbal Nur Iskandar:** Writing – review & editing, Investigation, Formal analysis, Data curation. **Melanie Melanie:** Writing – review & editing, Supervision. **Desak Made Malini:** Writing – review & editing, Supervision. **Camelia Panatarani:** Writing – review & editing, Supervision, Project administration. **I Made Joni:** Writing – review & editing, Supervision, Project administration. **Dedat Prismantoro:** Writing – review & editing, Visualization. **Febri Doni:** Writing – review & editing, Supervision. **Ravindra Chandra Joshi:** Writing – review & editing. **Wawan Hermawan:** Writing – review & editing, Supervision, Project administration, Funding acquisition.

## Declaration of competing interest

The authors declare that they have no known competing financial interests or personal relationships that could have appeared to influence the work reported in this paper.

## Data Availability

Data will be made available on request.
